# Diversification during cross-kingdom microbial experimental evolution

**DOI:** 10.1038/s41396-023-01479-w

**Published:** 2023-07-31

**Authors:** Ákos T. Kovács

**Affiliations:** Institute of Biology, Leiden University, 2333 BE Leiden, The Netherlands

**Keywords:** Microbial ecology, Microbial ecology

## Abstract

Experimental evolution in a laboratory helps researchers to understand the genetic and phenotypic background of adaptation under a particular condition. Simultaneously, the simplified environment that represents certain aspects of a complex natural niche permits the dissection of relevant parameters behind the selection, including temperature, oxygen availability, nutrients, and biotic factors. The presence of other microorganisms or a host has a major influence on microbial evolution that often differs from the adaptation paths observed in response to abiotic conditions. In the current issue of the ISME Journal, Cosetta and colleagues reveal how cross-kingdom interaction representing the cheese microbiome succession promotes distinct evolution of the food- and animal-associated bacterium, *Staphylococcus xylosus*. The authors also identified a global regulator-dependent adaption that leads to evolved derivatives exhibiting reduced pigment production and colony morphologies in addition to altered differentiation phenotypes that potentially contribute to increased fitness.

Numerous previous studies have previously explored evolution of microorganisms in a host or using a simplified host-mimicking environment. These studies advanced our understanding of how microorganisms adapt during host colonization and how specific genetic changes in the microbial genomes might contribute to chronic infections [1]. Similarly, bacterial adaptation has been characterized during plant colonization in distinct bacterial species [2, 3]. However, examples of multi-species experimental evolution approaches in natural or food-related environments are scarce. Cheese rind-derived microbial communities offer a tractable system to scrutinize microbial interactions that mimics the dynamics of cheese fermentation [4]. Furthermore, these simplified and sufficiently controlled communities facilitate the investigation of specific interactions among the members of a community, for example through transcriptional, and chemical characterization [5], and allow experimental evolution to dissect the genomic changes [6]. Cosetta et al investigated the evolution of *Staphylococcus xylosus* in the presence of the yeast *Debaryomyces hansenii*, the bacterium *Brevibacterium aurantiacum*, and the mold *Penicillium solitum* that all naturally co-occur during cheese rind community succession in a specific temporal order, the latter two microorganisms being abundant during later stages of rind development (Fig. 1) [4]. Intriguingly, experimental evolution of *S. xylosus* in the presence of *Debaryomyces*, unlike with *Brevibacterium* or *Penicillium*, is accompanied by the appearance of distinct morphotypes exhibiting reduced pigment production and colony architecture complexity [7]. Sequencing of evolved *S. xylosus* derivatives identified highly parallel mutations specific to repeated co-culture with *Debaryomyces*, mapped in global regulators of staphylococci including the alternative sigma factor B (SigB), the components of the quorum sensing Agr system, and the cell wall metabolism-related WalKR two-component system. Acquisition of mutations impeding the activity of SigB seems to be a generic adaption pathway in staphylococci: *Staphylococcus aureus* rapidly diversifies during biofilm formation in the presence of elevated magnesium as well as within an in vivo infection mouse model accompanied by loss of function SigB activity [8]. The evolved derivatives of *S. xylosus* with *sigB* mutations exhibit diminished pigment production and reduced synthesis of the carotenoid staphyloxanthin [7]. Although the reduced staphyloxanthin level weakens protection against oxidative stress caused by hydrogen peroxide, *sigB* mutation in the evolved *S. xylosus* derivatives enhances biofilm formation. However, increased biofilm development with diminished SigB activity appears to be species or strain dependent, as *S. aureus sigB* mutant displays reduced biofilm formation [8]. SigB has been demonstrated to influence the Agr quorum sensing system in staphylococci, which also acquired mutation in *S. xylosus* when co-evolving with *Debaryomyces*. The evolved isolates carrying mutations in the *agr* operon displayed increased biofilm formation, as expected based on other *Staphylococcus* species. Additionally, several evolved derivatives, especially those carrying an *agr* mutation, displayed reduced colony spreading [7]. It can be hypothesized that these mutations in *S. xylosus* might be selected in cheese rind microbiome at certain stages of community maturation, scrutinizing the metagenome of a cheese rind microbiome succession might reveal how much these simplified, laboratory co-cultures mirror the natural food fermentation process.

**Fig. 1:**
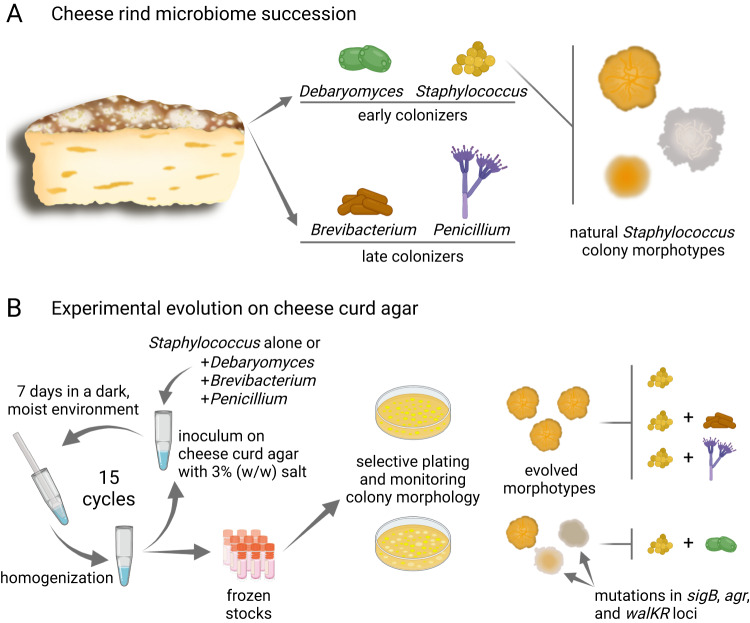
Natural succession and laboratory evolution of microbial interactions on cheese rind. Panel **A** depicts the cheese rind microbiome, including the early colonizer *Staphylococcus xylosus* and *Debaryomyces hansenii*, and the late successive *Brevibacterium aurantiacum* and *Penicillium solitum*. *S. xylosus* isolates display variable colony morphologies (morphotypes), including reduced structure and pigmentation. Panel **B** presents a laboratory experimental evolution with *S. xylosus* cultivated for 15 consecutive cycles alone or in co-culture with either *Debaryomyces*, *Brevibacterium*, or *Penicillium* on cheese curd agar medium. Co-evolution with the yeast, *Debaryomyces* was accompanied by *S. xylosus* morphotypes that displayed reduced colony architecture and carotenoid staphyloxanthin production. The evolved strains harbored mutations in one or several genes encoding a global transcriptional regulator, including the *sigB*, *agr*, and *walKR* loci. Created with BioRender.com.

The genetic and phenotypic adaptations of *S. xylosus* when co-evolved with *Debaryomyces* are accompanied by an increased fitness of this bacterium in co-cultures with the yeast, but not in the presence of other cheese-derived bacterial and mold isolates, *B. aurantiacum* and *P. solitum*. Moreover, one of the *Debaryomyces*-adapted *S. xylosus* clones even displays reduced fitness in competition with *Brevibacterium* and *Penicillium*. These results support and mimic the appearance of pigment-reduced *Staphylococcus* isolates during natural cheese fermentation, potentially driven by direct biotic interaction with the yeast or the abiotic niche created by the yeast. However, a subsequent increase of *Brevibacterium* and *Penicillium* during the later stages of cheese rind community succession could potentially restrain the pigment-reduced *Staphylococcus* clones. The impact of *Brevibacterium* and *Penicillium* on the fitness of the *Debaryomyces* co-adapted *S. xylosus* strains with acquired mutations in global regulators questions whether such evolution trajectory would be suppressed in a synthetic community containing all microbial members.

The exact mechanism of how *Debaryomyces* promotes diversification of *S. xylosus* on cheese and the trigger of bacterial fitness benefit in co-cultures with yeast remain enigmatic. Similarly, laboratory adaptation of another Gram-positive bacterium, *Bacillus subtilis* to the presence of a fungus is similarly entailed by mutations in a global transcriptional regulator [9]. In that example, an elevated level of antifungal lipopeptide by the evolved derivatives creates cell wall stress in the fungus and increases the fitness of the bacterium by colonizing its niche. Next steps should focus on revealing how the interaction between *S. xylosus* and *Debaryomyces* adjusts after their co-adaptation, whether metabolic interplay changes, the bacterial cells acquire higher resistance to the yeast, or *Staphylococcus* inhibits *Debaryomyces*. As Cosetta and colleagues highlight, niche overlap could potentially be a strong promotor of the diversification of *S. xylosus*. Moreover, global transcriptional profiling of the yeast co-evolved *S. xylosus* clones revealed the downregulation of various pathways connected to the biosynthesis of amino acids, which is hypothesized to work in concert with the released nutrients derived from *Debaryomyces*. Metabolic and transcriptional characterization of the evolved bacterium and yeast in co-cultures might disclose the intricate co-evolution between these two microorganisms.

In summary, the study of Cosetta and colleagues teaches us how biotic environments, here specifically the repetitive co-cultivation of microorganisms influence microbial adaption. The study also demonstrates the parallelism in phenotypic changes of microorganisms observed during food fermentation process and laboratory experimental evolution, and therefore help us learn about how microorganisms may evolve in complex environments (Fig. 1).
