# Correction: Diversification during cross-kingdom microbial experimental evolution

**DOI:** 10.1038/s41396-023-01551-5

**Published:** 2023-11-15

**Authors:** Ákos T. Kovács

**Affiliations:** Institute of Biology, Leiden University, 2333 BE Leiden, The Netherlands

**Keywords:** Microbial ecology, Microbial ecology

Correction to: *The ISME Journal* 10.1038/s41396-023-01479-w, published online 31 July 2023

The article “Diversification during cross-kingdom microbial experimental evolution“ written by Ákos T. Kovács,was originally published Online First without Open Access. After publication in volume 17, issue 9, page 1355–1357 the author decided to opt for Open Choice and to make the article an Open Access publication. Therefore, the copyright of the article has been changed to © [copyright holder name in OA Team’s request] [year of online publication] and the article is forthwith distributed under the terms of the Creative Commons Attribution 4.0 International License, which permits use, sharing, adaptation, distribution and reproduction in any medium or format, as long as you give appropriate credit to the original author(s) and the source, provide a link to the Creative Commons licence, and indicate if changes were made. The images or other third party material in this article are included in the article’s Creative Commons licence, unless indicated otherwise in a credit line to the material. If material is not included in the article’s Creative Commons licence and your intended use is not permitted by statutory regulation or exceeds the permitted use, you will need to obtain permission directly from the copyright holder. To view a copy of this licence, visit http://creativecommons.org/licenses/by/4.0.

